# Fine mapping of the sex locus in *Salix triandra* confirms a consistent sex determination mechanism in genus *Salix*

**DOI:** 10.1038/s41438-020-0289-1

**Published:** 2020-05-01

**Authors:** Wei Li, Huaitong Wu, Xiaoping Li, Yingnan Chen, Tongming Yin

**Affiliations:** The Key Lab of Cultivar Innovation and Germplasm Improvement of Salicaceae, College of Forestry, Nanjing Forestry University, Nanjing, 210037 China

**Keywords:** Plant breeding, Evolutionary biology

## Abstract

*Salix triandra* belongs to section *Amygdalinae* in genus *Salix*, which is in a different section from the willow species in which sex determination has been well studied. Studying sex determination in distantly related willow species will help to clarify whether the sexes of different willows arise through a common sex determination system. For this purpose, we generated an intraspecific full-sib F_1_ population for *S. triandra* and constructed high-density genetic linkage maps for the crossing parents using restriction site-associated DNA sequencing and following a two-way pseudo-testcross strategy. With the established maps, the sex locus was positioned in linkage group XV only in the maternal map, and no sex linkage was detected in the paternal map. Consistent with previous findings in other willow species, our study showed that chromosome XV was the incipient sex chromosome and that females were the heterogametic sex in *S. triandra*. Therefore, sex in this willow species is also determined through a ZW sex determination system. We further performed fine mapping in the vicinity of the sex locus with SSR markers. By comparing the physical and genetic distances for the target interval encompassing the sex determination gene confined by SSRs, severe recombination repression was revealed in the sex determination region in the female map. The recombination rate in the confined interval encompassing the sex locus was approximately eight-fold lower than the genome-wide average. This study provides critical information relevant to sex determination in *S. triandra*.

## Introduction

Flowering plants have evolved a considerably more complex sex determination system than animals, which have distinct germlines. Sexual diversity can first be observed in the various floral forms, ranging from hermaphroditic to monoecious and dioecious or even trioecious^1^. Approximately 5–10% of angiosperms are dioecious, with either heteromorphic or homomorphic sex chromosomes^2^. All sex chromosomes in dioecious plants harbor a sex determination region (SDR), which is characterized by suppressed recombination, leading to haplotype divergence^3^. Pseudo-autosomal regions (PARs) are also present in plant sex chromosomes, with the physical lengths of PARs varying among different plants^4^. Different sex determination systems, including male heterogamety (XY system) and female heterogamety (ZW system), have been described in dioecious plants^5^. The overall interactions among elements such as plant hormones, genetic factors, and epigenetic modifications determine plant sex^6^.

*Populus* and *Salix* are sister genera in the Salicaceae family. These two lineages diverged at least 45 million years ago^7,8^. The chromosome number of willows and poplars is the same, and they do not exhibit a morphologically differentiated sex chromosome^9,10^. The Salicaceae family, whose young sex chromosomes have evolved from different autosomes, provides a valuable comparative system for studying sex differentiation in plants^11^. Multiple studies have reported that chromosome XIX is the incipient sex chromosome in poplars^12^, and both XX/XY^13,14^ and ZW/ZZ^12,15^ sex determination systems have been observed in different poplar species. In poplars, the sex-determining locus has been mapped to either the peritelomeric region^12^ or the centromeric region of chromosome XIX^14,16^. However, no sex-determining genes have been identified among the *Populus* species analyzed so far, and only candidate genes have been reported. For example, the male-specific *TOZ19* gene was identified as a sex determination candidate in *P. tremula* and *P. tremuloides*^17^. The sex determination region in poplar was found to contain a large number of *R* genes^12,18^, and thus, it was hypothesized that the emergence of the sex determination region might have been due to selective pressure arising from sex-specific floral pathogens^19^. In *P. balsamifera*, the *PbRR9* gene exhibits male-biased methylation, indicating a role of epigenetic regulation in poplar sex determination^20^.

In willows, the sex determination locus has been consistently mapped to chromosome XV, and only the ZW sex determination system has been observed^21–24^. The reconstruction of alternate haplotypes in the SDR revealed sequence divergence between the Z and W chromatids^22^, and no homologous genes in the SDR have been found between the willow and poplar^22,23^. Pucholt et al.^23^ localized the sex determination locus to a 2.5-Mb genomic region in *S. viminalis* that harbors 48 protein-coding genes. Further study showed that the SDR in *S. viminalis* is of limited size (~804 kb) and exhibits a higher SNP density in females^25^. Pseudogenization and the accumulation of repetitive elements in the SDR suggest that the fundamental process of sex chromosome formation occurred very swiftly after recombination ceased^11^. In a recent study, the SDR of *S. purpurea* was found to contain large palindromic repeats, and the *SpRR9* gene was considered a putative candidate for controlling sex determination through the modulation of the cytokinin signaling pathway^26^. Whether willow exhibits a relatively conserved sex determination system needs to be explored in more willow species.

*S. triandra* is a shrub willow belonging to section *Amygdalinae* in genus *Salix*. It is distributed widely from Japan to western Europe^27^. More recently, *S. triandra* has received attention because of its potential implications in insect resistance^28,29^. Due to the reproductive efficiency, easy cultivation, and small genome, *S. triandra* is suitable for obtaining additional information to better understand sex determination in dioecious plants. In this study, *S. triandra* is used to provide new evidence of the sex determination mechanism in willow. Our purpose is to clarify whether the previously reported willow sex determination system also functions in a willow species belonging to a different section of genus *Salix*.

## Materials and methods

### Plant materials and DNA extraction

The mapping population, which consisted of 152 full-sib F_1_ progenies, was established in 2013 by crossing the *S. triandra* female clone “DB447” with the male clone “DB134”. “DB447” and “DB134” were sampled from the Maoer Mountain in Heilongjiang Province of China (permissions were granted by the local administration). The parental clones and progeny were maintained at the Baima Forest Farm in Lishui in Jiangsu Province, China. Genomic DNA was extracted from the young leaves of each individual by using an E.Z.N.A. Plant DNA Kit (Omega Bio-tek, Norcross, GA, USA). DNA quality was assessed by 1% agarose gel electrophoresis, and the DNA concentration was measured with a Nanodrop 2000 (Thermo Scientific, MA, USA).

### Library construction and sequencing

The whole-genome sequencing (WGS) was conducted with the two crossing parents, and restriction site-associated DNA (RAD) sequencing was performed for 152 progenies of the mapping population. For the crossing parents, two paired-end libraries with 300–500 bp insert sizes were constructed separately according to the standard protocol of Illumina (Illumina). For each progeny, the RAD library was prepared following the method described by Baird et al.^30^ with minor modifications. Briefly, 300 ng of genomic DNA from each progeny was digested separately by using 5 U of Tap I (Takara Bio, Japan) at 37 °C for 60 min, and then the P1 adapter, which contained a forward primer site, an Illumina sequencing primer site and a barcode (4–8 bp), was ligated to the fragments. Subsequently, the P1-ligated fragments of all samples (1 µL each) were pooled and then randomly sheared (Bioruptor) to an average size of 500 bp.

The entire sample was separated using 1% agarose gel electrophoresis, and the DNA fraction corresponding to 300–700 bp was isolated using an E.Z.N.A. Gel Extraction Kit (Omega Bio-tek, USA). The purified fragments were subjected to end repair and the 3′-end addition of dATP overhangs, followed by the ligation of a P2 adapter containing a reverse primer site and an Illumina sequencing primer site. Finally, the RAD library was selectively enriched by PCR amplification with the P1-forward primer and P2-reverse primer, and the 300–700 bp amplicons were purified again with the Gel Extraction Kit (Omega Bio-tech, USA).

Both WGS and RAD sequencing were performed on the Illumina HiSeq X Ten platform (Illumina, USA) at Shanghai Major Biological Medicine Technology following the manufacturer’s protocol (Illumina).

### Sequence analysis and nucleotide variant identification

Raw reads were assigned to each individual based on the unique barcodes and then subjected to quality control, adapter trimming and read filtering by using FASTP (version 0.6.0, https://github.com/OpenGene/fastp). Reads that contained >40% low-quality bases (base quality value <15) or >10% N bases were discarded. Sequences shorter than 30 bp after trimming were also removed.

The resulting high-quality reads were mapped to the reference genome of *S. purpurea* v1.0 (DOE-JGI, http://phytozome.jgi.doe.gov/pz/portal.html#!info?alias= Org_Spurpurea) by using BWA (version 0.7.16, http://bio-bwa.sourceforge.net/) software^31^ with the default parameters. GATK Haplotype Caller^32^ was used to call nucleotide variants, including SNPs and InDels, which were further filtered according to GATK Best Practices^33^.

### Linkage map construction

To obtain high-quality linkage maps, all the filtered genetic markers were further screened based on the following criteria: (1) average sequence depth >5× in the parents and >4× in the progeny; (2) heterozygous as least in one parent; (3) present in ≥70% progeny; and (4) following a Mendelian segregation ratio. Markers with significant segregation distortion (*χ*^2^ test, *P* < 0.05) were excluded from linkage map construction.

The integrated linkage analysis was performed by using JoinMap 5.0 (https://www.kyazma.nl/index.php/JoinMap/), and a logarithm of odds (LOD) score threshold of 4.0 was employed to establish linkage groups (LGs). The female and male maps were constructed with a two-way pseudo-testcross strategy. The LGs were nominated according to the alignment of the mapped markers with the *S. purpurea* v1.0. genome assembly. The genetic distance between markers was estimated using the Kosambi mapping function^34^. The marker distribution in each LG was analyzed using the sliding window (10 cM) approach^35^. The quality of the genetic map was assessed using a haplotype map and a heat map^36^.

### Linkage analysis of the sex locus

The sex of the plants was visually recorded for the 152 progenies. Among these progenies, 77 were female and 75 were male. The phenotypic data were included in the data matrix of each parent and scored as a testcross marker. Based on the established genetic maps, the sex locus was mapped as a segregating morphological marker with MapMaker software (version 3.0). To verify the accuracy of the positioning interval, we designed SSR markers with a physical distance of 4 Mb upstream and downstream from two SNP markers that were completely linked with sex.

## Results

### DNA sequencing data

**Fig. 1:**
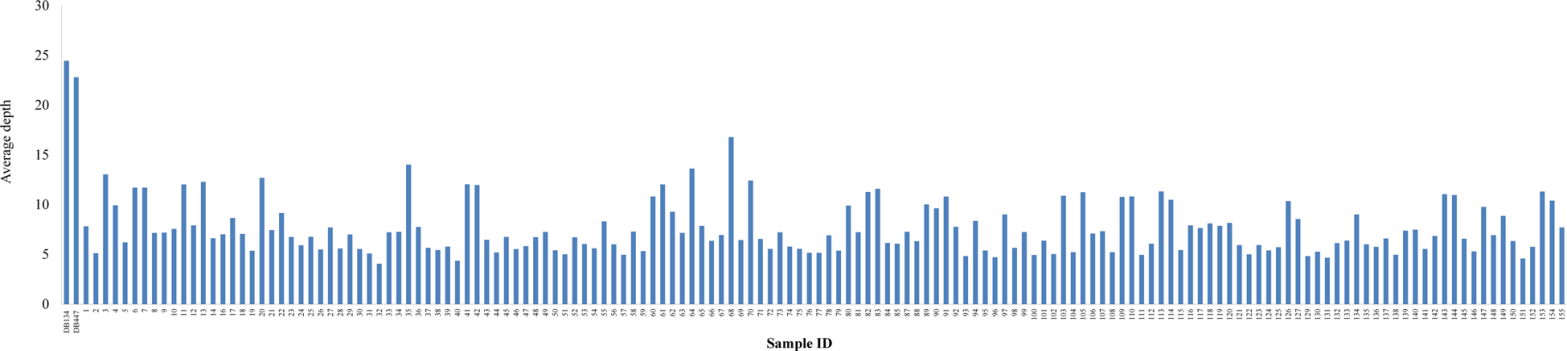
Sequencing depth of each sample. The sampled accessions are indicated on the *x*-axis

For each parent, WGS yielded 9.65 Gb of clean sequencing data on average, and the sequencing depth was ~20× genome coverage (Fig. 1). After quality control, a total of 136.96 M clean reads were obtained from the two parents, with an average Q30 ratio of 90.03% and an average guanine-cytosine (GC) content of 37.25% (Table 1). For the 152 F_1_ offspring, a total of 504.21 Gb of clean data were generated, including 3622.24 M of high-quality clean reads with a length of 150 bp (Table 1). The number of the clean reads ranged from 11.03 to 53.06 M among different offspring, with an average of 23.83 M. The average sequencing depth for each progeny was approximately 7.53×, varying from 4.06× to 16.78× (Fig. 1). The average Q30 ratio was 88.88%, and the GC content was 41.58% (Table 1).

**Table 1:** Statistics of the WGS and RAD sequencing results in *S. triandra*

Sample	Raw data (Gb)	Clean data (Gb)	Clean reads (M)	Clean data percentage (%)	Q30 (%)	GC (%)
DB134	10.94	9.90	70.07	90.49	90.43	36.40
DB447	10.49	9.41	66.89	89.70	89.63	38.10
Progeny	603.69	504.21	3622.24	83.52	88.88	41.58
Total	625.12	523.52	3759.20	—	—	—

### Nucleotide variant discovery and genotyping

The high-quality reads obtained from all samples were separately mapped to the reference genome of *S. purpurea* v1.0, and the mapped ratio ranged from 46.89% to 88.04%, with an average of 80.34% (Supplementary Table S1). All mapped reads were used for SNP calling, and a total of 1,150,885 putative nucleotide variant loci were detected in both parents. Based on genotyping information and the stringent filtering criteria described in the ‘Materials and methods' section, 22,830 high-quality markers were retained from the whole F_1_ population, including 20,695 SNPs and 2135 InDels (Table 2). Among these markers, 9188 (8301 SNPs and 887 InDels) were only maternally informative (nn×np), 9089 (8297 SNPs and 792 InDels) were only paternally informative (lm×ll), and the remaining 4553 (4097 SNPs and 456 InDels) were intercrossing markers in both parents (Table 2).

**Table 2:** Summary of the marker types and numbers of markers used for genetic map construction

Marker type	SNP number	InDel number
ef×eg (1:1:1:1)	6	2
hk×hk (1:2:1)	4091	454
lm×ll (1:1)	8297	792
nn×np (1:1)	8301	887
Total	20,695	2135

Note: The ‘lm×ll’ and ‘nn×np’ segregation types represent markers that are heterozygous only in the paternal or maternal parent, respectively.

### Construction and evaluation of the high-density linkage map

We constructed the paternal, maternal and consensus maps for *S. triandra* separately. The maternal map was 2193.78 cM in length, with LG sizes ranging from 91.25 cM (LG13) to 141.64 cM (LG14) (Table 3). The paternal map was 2381.93 cM in length, with LG sizes varying from 90.34 cM (LG17) to 148.70 cM (LG1) (Table 3).

**Table 3:** Summary of the linkage map of *S. triandra*

Maternal map			Paternal map			Consen**s**us map		
Group	Total marker	Total distance (cM)	Group	Total marker	Total distance (cM)	Group	Total marker	Total distance (cM)
FLG1	837	118.00	MLG1	805	148.70	LG1	1355	143.26
FLG2	788	132.74	MLG2	882	124.02	LG2	1402	142.75
FLG3	725	136.62	MLG3	713	137.28	LG3	1183	103.54
FLG4	481	101.33	MLG4	549	136.98	LG4	869	142.71
FLG5	782	128.83	MLG5	793	104.40	LG5	1296	110.57
FLG6	872	129.78	MLG6	794	125.72	LG6	1387	96.87
FLG7	602	138.12	MLG7	618	140.52	LG7	998	102.74
FLG8	729	113.96	MLG8	667	136.27	LG8	1164	140.66
FLG9	524	91.60	MLG9	576	125.62	LG9	912	98.54
FLG10	775	91.70	MLG10	827	107.81	LG10	1377	101.10
FLG11	800	129.95	MLG11	827	128.47	LG11	1353	116.95
FLG12	512	127.17	MLG12	612	103.98	LG12	967	97.31
FLG13	770	91.25	MLG13	719	117.28	LG13	1231	129.91
FLG14	491	141.64	MLG14	527	123.03	LG14	874	145.29
FLG15	810	105.68	MLG15	713	133.58	LG15	1269	125.36
FLG16	1401	110.86	MLG16	1432	123.74	LG16	2314	144.95
FLG17	702	105.81	MLG17	725	90.34	LG17	1170	139.85
FLG18	667	96.65	MLG18	580	132.16	LG18	1094	120.19
FLG19	374	102.09	MLG19	382	142.02	LG19	615	137.16
Total	13,642	2193.78	Total	13,741	2381.93	Total	22,830	2239.71

**Fig. 2:**
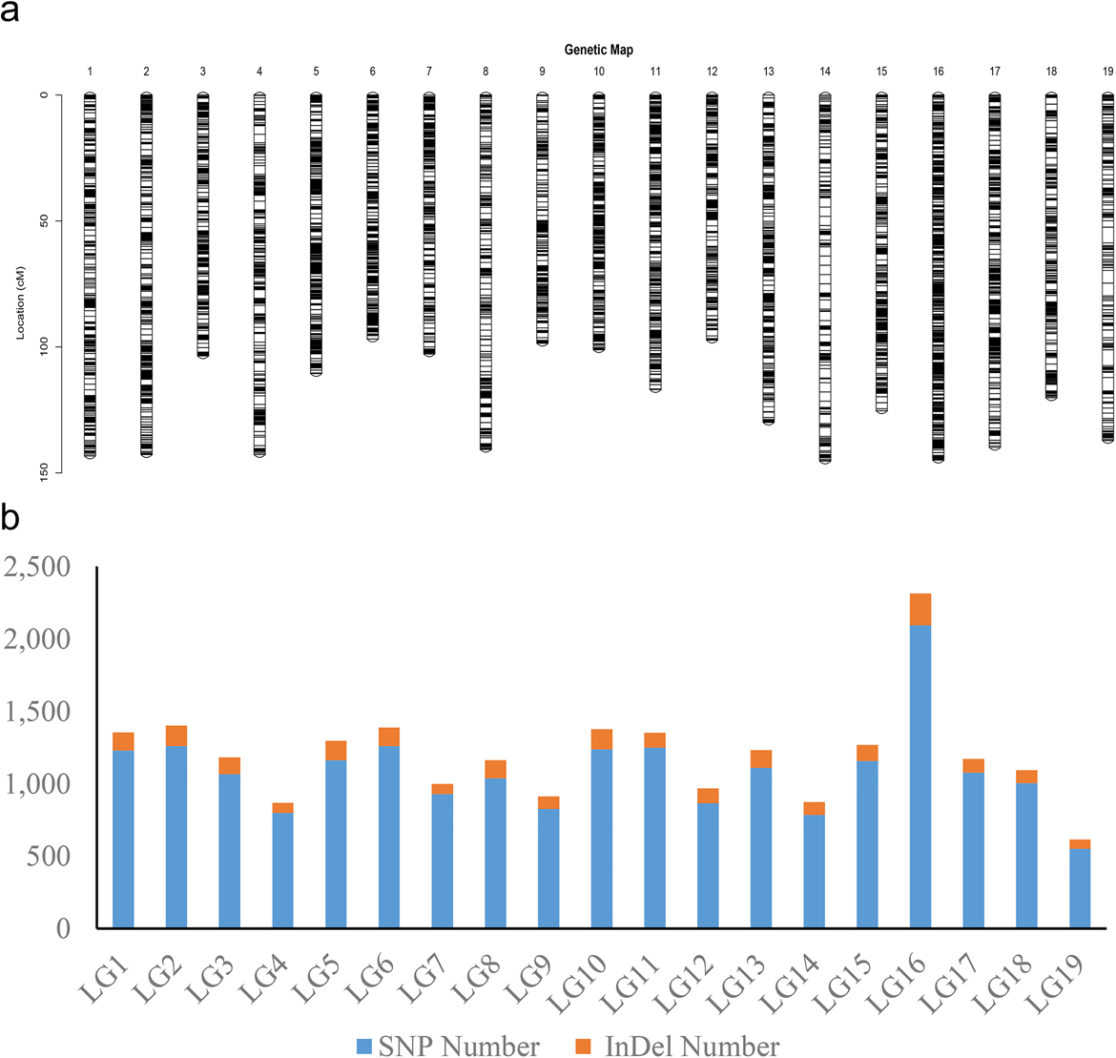
The consensus genetic map of *S. triandra*. **a** Distribution of mapped markers within each linkage group of *S. triandra*. A black line indicates a SNP/InDel marker. **b** The number of SNPs and InDels in each linkage group

**Fig. 3:**
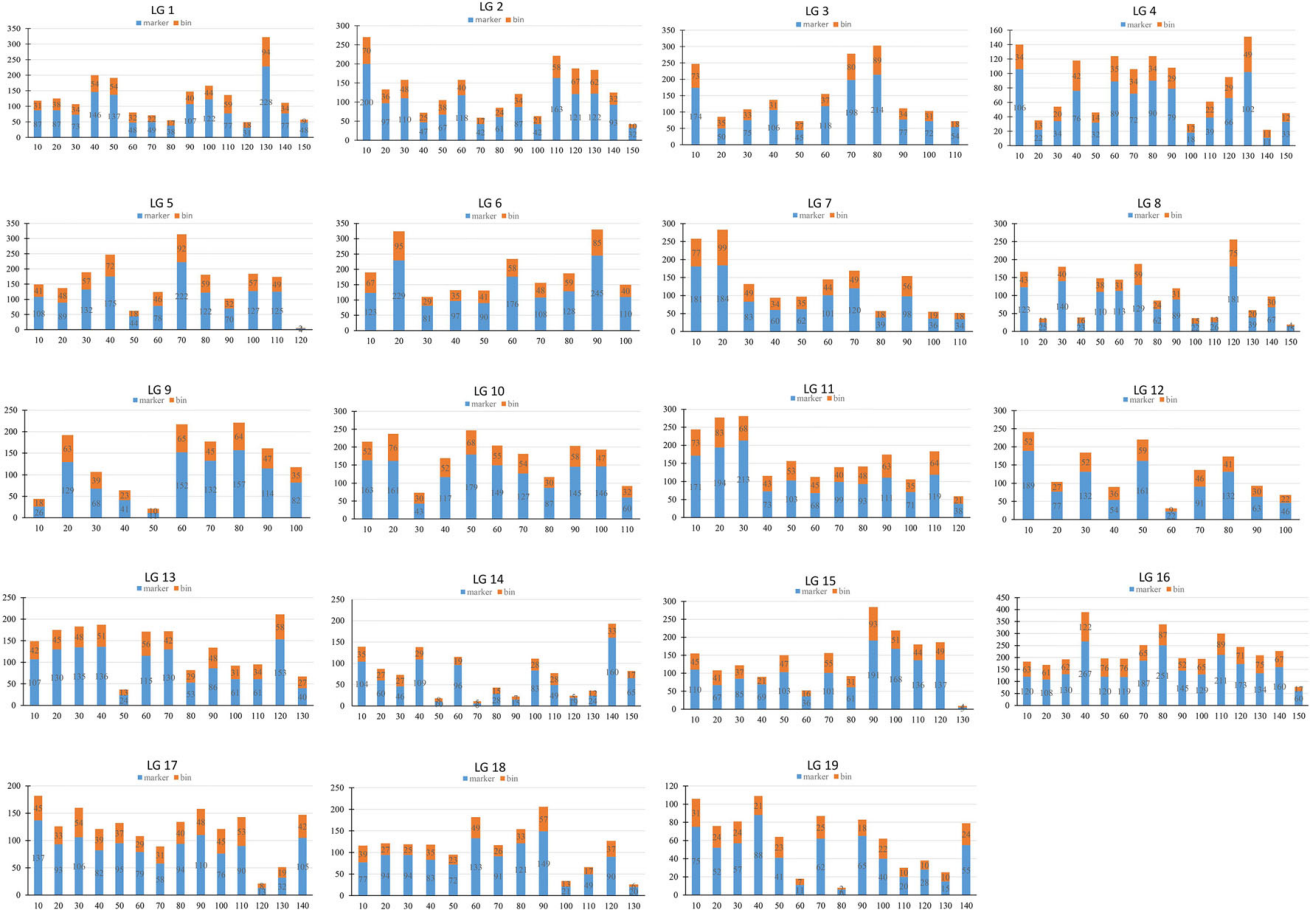
Marker distribution analysis of the consensus map. The numbers of markers and marker bins are indicated with blue and yellow bars, respectively

All 22,830 markers that segregated as ef×eg, hk×hk, lm×ll or nn×np were used to generate a consensus map for *S. triandra*. At the LOD threshold of 4.0, all of these markers were successfully grouped into 19 LGs (Fig. 2 and Fig. S1). The number of LGs was consistent with the haploid chromosome number of willows (2*n* = 38). The established consensus map covered a genetic distance of 2239.71 cM, with LG sizes varying from 96.87 cM (LG6) to 145.29 cM (LG14) (Table 3). The marker distribution along each LG was evaluated by counting the number of marker bins and all mapped markers using a sliding window of 10 cM. The average number of marker bins ranged from 18.00 to 69.87, with the average number of mapped markers varying from 43.93 to 154.27. The window with the highest density (26.7 markers per cM) was found in LG16 (Fig. 3).

Haplotype maps, which revealed the missing data and recombination events of each individual intuitively, were generated for each LG in the 152 offspring (Supplementary Fig. S2). The percentages of missing data and double crossovers were less than 0.30% and 0.23%, respectively. Based on the pairwise recombination values of the markers grouped in each LG, heat maps were generated for the 19 LGs (Supplementary Fig. S3). All the heat maps demonstrated a clear trend in which the pairwise linkage generally decreased with an increase in genetic distance between the mapped markers, indicating that the markers in each LG were precisely mapped and ordered.

### Collinearity between the genetic map and reference genome

**Fig. 4:**
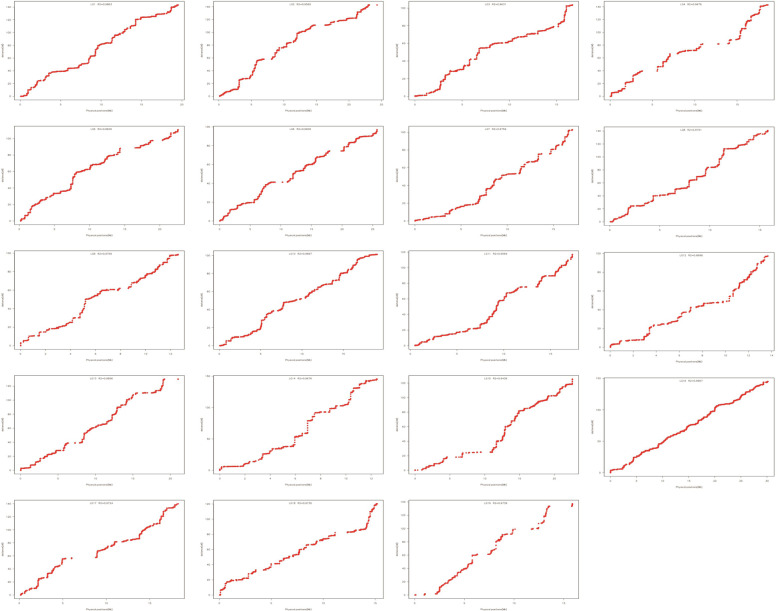
Collinearity analysis between the integrated *S. triandra* genetic map and the *S. purpurea* v1.0. genome. The marker position in the genome is shown on the *x*-axis; the marker position in each linkage group is shown on the *y*-axis

All the mapped markers were aligned to the *S. purpurea* v1.0 genome to estimate the physical distances of the markers and to assess the collinearity between the genetic map and reference genome. In general, high collinearity was observed between the markers and the corresponding chromosomes (Fig. 4), with the Spearman rank correlation coefficient ranging from 0.99–1.00. However, there were also LGs showing discrepancies in some narrow regions (e.g., LGs 14, 18 and 19), which might have been due to different recombination rates, missing data or compromised marker orders in the consensus map.

The mapped markers in the integrated genetic map covered at least 96.95% of the physical length of the reference genome (Table 4). The genetic-to-physical distance ratios ranged from 3.78 cM/Mb (LG6) to 11.65 cM/Mb (LG14) (Table 4). The marker density along each chromosome ranged from 28.81 to 70.64 markers/Mb, averaging 49.18 markers/Mb (Table 4).

**Table 4:** Statistics of the collinearity analysis between the integrated *S. triandra* genetic map and the *S. purpurea* v1.0. genome

LG	Physical coverage (%)	Genetic distance/physical distance (cM/Mb)	Density (markers/Mb)	Spearman correlation
1	99.54	7.35	55.10	0.99
2	99.34	5.91	65.73	1.00
3	99.85	6.19	57.08	0.99
4	99.72	7.67	42.89	0.99
5	98.75	4.93	33.47	1.00
6	99.63	3.78	28.81	1.00
7	99.78	6.03	35.61	1.00
8	98.98	8.94	53.54	0.99
9	99.21	7.83	40.35	0.99
10	99.62	5.32	46.90	1.00
11	96.95	6.95	65.70	1.00
12	99.38	7.12	51.26	0.99
13	99.87	6.18	42.68	0.99
14	99.75	11.65	60.42	0.99
15	99.91	5.55	42.30	1.00
16	99.96	4.81	70.64	1.00
17	99.11	7.73	58.53	0.99
18	99.62	7.93	52.38	0.99
19	99.76	8.66	31.05	0.99
Mean	99.41	6.87	49.18	0.99

### Mapping the sex locus and gene content in the confined genetic interval

**Fig. 5:**
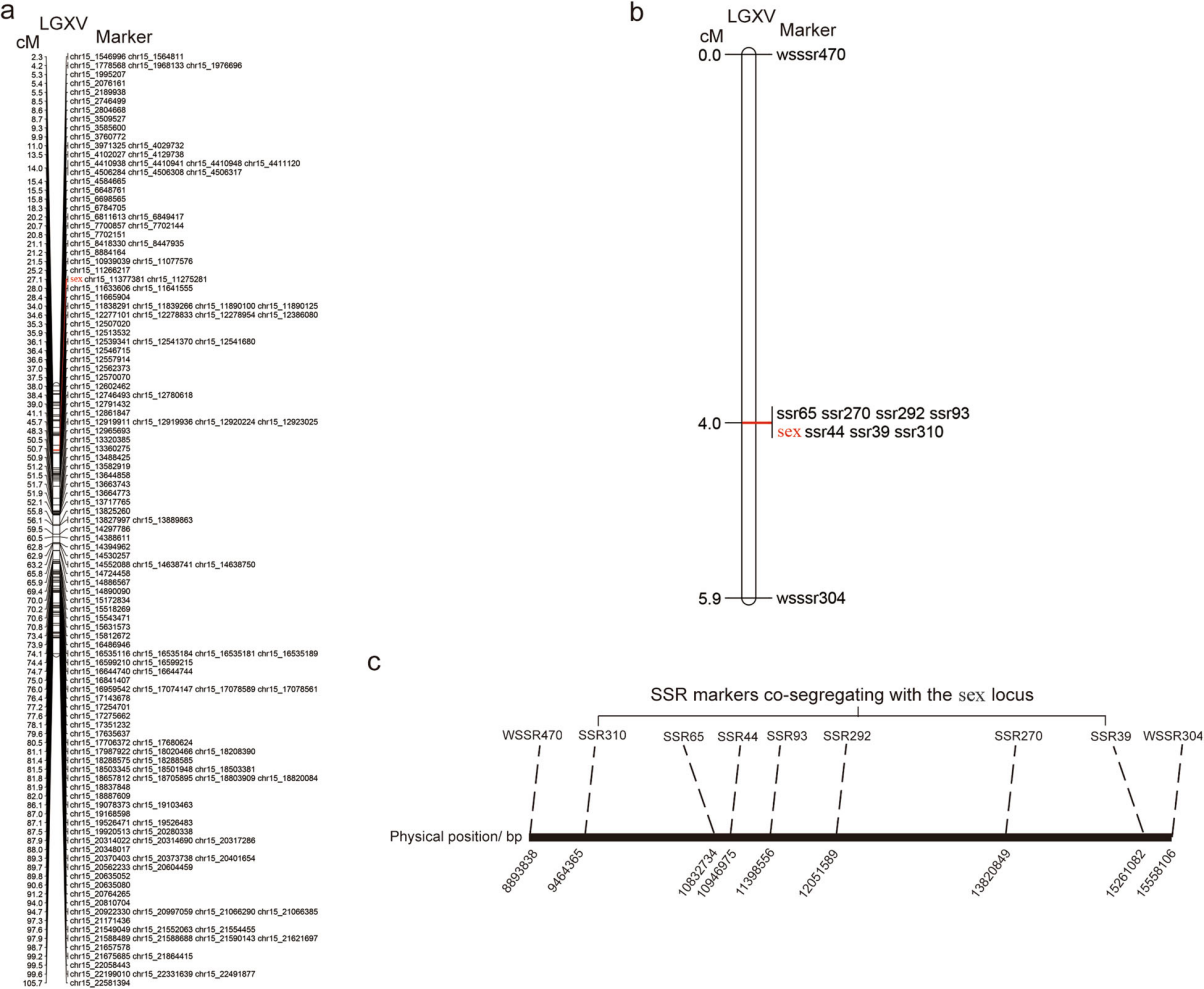
Locating the sex locus of *S. triandra*. **a** Linkage analysis mapping the sex locus of *S. triandra* within LGXV of the female map. **b** Fine positioning of the SSR markers in the vicinity of the sex locus in *S. triandra*. **c** The physical positions of SSR markers in the confined IESL

The mapping results showed that the sex locus could only be mapped in the maternal map 27.07 cM from the telemetric end of chromosome XV (Fig. 5a), and no linkage with sex was detected in the paternal map. Using the sequences of the SNP markers co-segregating with sex, we obtained genome sequences in the confined genetic interval and developed upstream and downstream sex-linked simple sequence repeat (SSR) markers. In total, seven SSR markers co-segregating with the sex locus were generated in the SDR on chromosome XV of the female (Fig. 5c), and the confined interval encompassing the sex locus (IESL) was bounded by SSR markers wssr304 and wssr470, with spacing of 5.9 cM (Fig. 5b). Based on the reference genome of *S. purpurea*, the confined IESL corresponded to a 6.5-Mb genomic region on chromosome 15. On average, a 1 cM genetic length contains a 140 kb sequence in the willow genome. Thus, the recombination rate in the confined IESL of the female is approximately eight-fold lower than the genome-wide average.

**Fig. 6:**
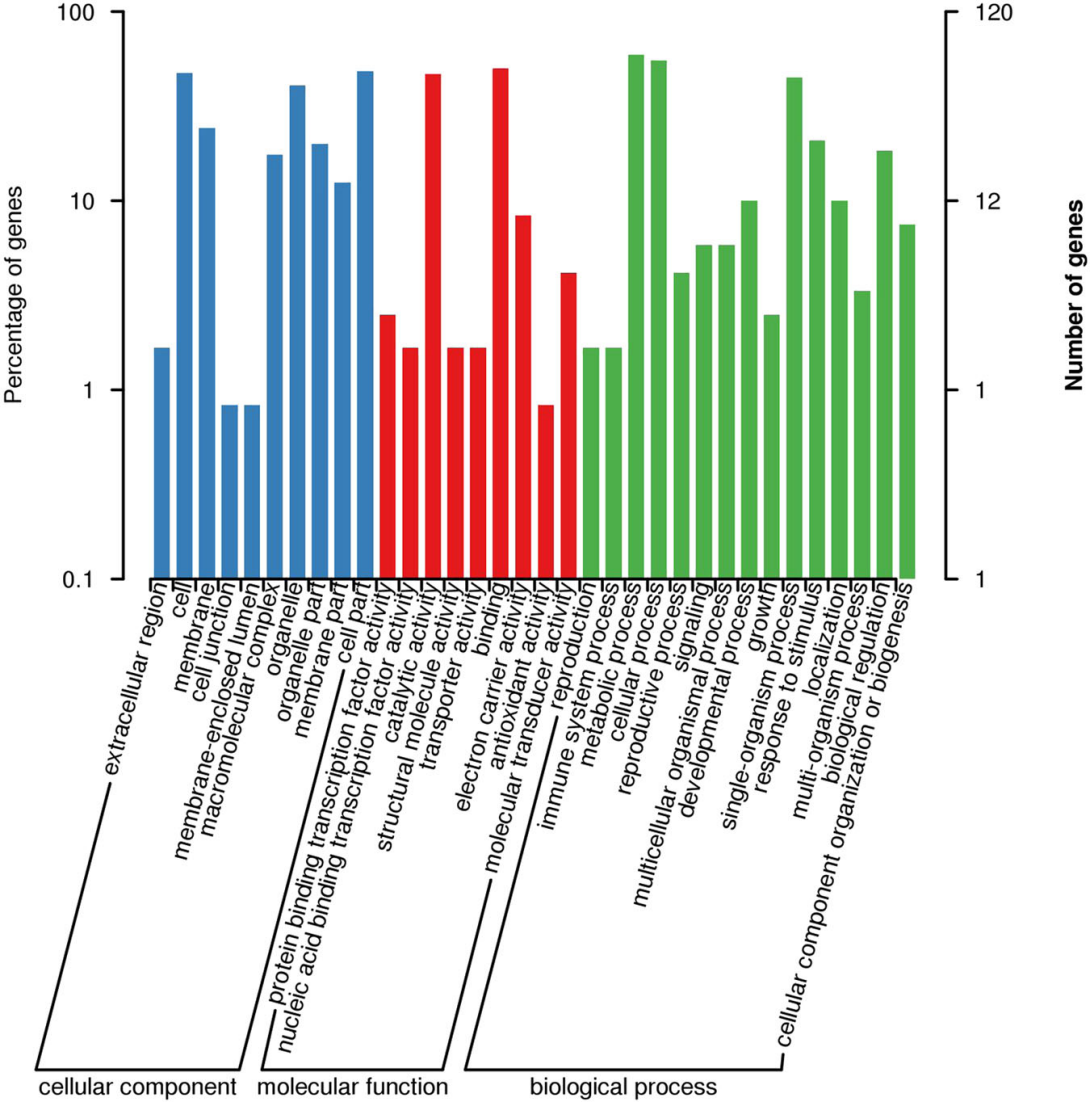
Numbers of willow unigenes in each functional category in the confined IESL in *S. triandra*

The target region harbored 249 genes. Gene Ontology (GO) terms clarified that genes involved in metabolic processes, cellular processes, single organism processes, reproductive processes and biological organization were the most represented groups (Fig. 6). Six genes were associated with microtubule motor activity (GO:0003777), and six genes were associated with microtubule binding activity (GO:0008017). EVM0038350 has a methyltransferase domain that may be related to DNA methylation. Salix_newGene_2 is a homologous gene of LRR receptor-like serine/threonine-protein kinase ERL1 in *Arabidopsis thaliana*, which is important for anther lobe formation. The *EVM0005130* gene contains a Myb-like DNA-binding domain that plays an important role in regulating anther and pollen development; EVM0045351 contains a mitogen-activated protein kinase (MAP kinase) domain that may function as a regulator of pollen development and germination.

In addition to the genes involved in flower organ development, flower development-associated miR156 was also found in the confined IESL. miR156 has emerged as the most important regulator in the vegetative phase change and the vegetative-to-reproductive transition in both Arabidopsis and maize. The candidate genes related to flower development are listed in Table 5.

**Table 5:** Classification and function of important genes in the confined IESL in chromosome XV of *S. triandra*

Gene ID	Start	End	Strand	Function distribution annotation
EVM0002598	12,380,227	12,385,512	−	Microtubule motor activity
EVM0006532	13,615,059	13,624,340	−	Microtubule motor activity
EVM0048283	13,638,084	13,638,395	−	Microtubule motor activity
Salix_newGene_14	14,399,846	14,402,709	+	Microtubule motor activity
Salix_newGene_6	13,351,098	13,354,415	+	Microtubule motor activity
Salix_newGene_7	13,354,476	13,357,328	+	Microtubule motor activity
EVM0038350	12,867,149	12,869,132	+	Methyltransferase activity
EVM0005130	13,655,785	13,658,118	−	Myb-like DNA-binding domain
Salix_newGene_28	14,013,632	14,016,788	−	RNA-directed RNA polymerase activity
EVM0008632	13,406,181	13,414,288	−	RNA processing and modification
EVM0045351	11,991,112	12,004,061	+	Mitogen-activated protein kinase (MAP kinase) domain
Salix_newGene_2	12,380,227	12,385,512	−	LRR receptor-like serine/threonine-protein kinase

## Discussion

The Salicaceae family is a valuable model system for revealing the origin and evolution of plant sex chromosomes. These genera are widely distributed around the globe, representing a diverse assemblage of subtrees, shrubs^37^ and catkin-bearing trees. Nearly all species in genera *Salix* (Ca. 500 species)^38^ and *Populus* (Ca. 30 species)^39^ are dioecious, though obvious heteromorphic sex chromosomes have not yet evolved. Furthermore, the two lineages share a well-preserved whole-genome duplication^7,8^ and show an ongoing propensity toward polyploid formation^40,41^, which facilitates the exploration of the relationship between polyploidy and sex chromosome evolution^42^.

In the past decade, there have been many reports on the sex determination mechanisms of poplars. In both *P. deltoides* and *P. nigra*, which are from section *Aigeiros*, the SDR is located at the proximal telomeric end of chromosome XIX^12,13^. In *P. tremuloides*, *P. tremula* and *P. alba*, all of which belong to section *Populus*, a pericentromeric region of chromosome XIX^14,15,43^ was determined to be the SDR. Both female heterogamety^12,15^ and male heterogamety have been reported^13,43^. Recently, sex determination was explored in 52 *P. trichocarpa* (section *Tacamahaca*) and 34 *P. balsamifera* (section *Populus*) individuals by using the genome-wide association study. A total of 650 sex-associated SNPs were found to be heterozygous in males, indicating an XY sex determination system in these two species^44^. In genus *Salix*, the SDR is confined to the centromeric region of chromosome XV in *S. viminalis* (section *Viminella*)^22,23,45^ and *S. suchowensis* (section *Helix*)^22,23,45^. Female heterozygosis predominates in the SDRs of these species, suggesting a ZW sex determination system in *Salix*. Furthermore, no candidate genes in the willow SDR are orthologous to those in the poplar SDR^22,23^. Current research is not sufficient to demonstrate whether the sex determination mechanisms of the two lineages are related.

In this study, we sought to explore the SDR in an additional willow species, *S. triandra* (section *Amygdalinae*). In the *S. triandra* SDR, obvious recombination suppression was observed in the female. Recombination suppression, which means that homologous chromosomes cannot pair and undergo recombination, is an important component of sex chromosome evolution^46^. In both advanced and primitive sex chromosomes, the suppression of recombination in chromosomal regions is observed in numerous plant species. It has been reported that both the X and Y chromosomes of *Actinidia chinensis var. chinensis* exhibit a similar pattern of restricted recombination: approximately one-third of the sex chromosome (terminal ~6 Mb of chromosome XXV) spans the SDR and shows severe recombination suppression, while the remaining section undergoes normal recombination^47^. Recombination suppression is also observed in the SDR in papaya, and the nonrecombining region of the Y chromatid differed greatly from the corresponding region of the X chromatid due to two large inversions^48^. In hop (*Humulus lupulus*), an approximately four-fold reduction in recombination is found on the Y chromosome compared with the X chromosome linkage map^49^. A study on *Silene alba* also found that almost the entire SDR of the sex chromosome showed heavily suppressed recombination^50^. Chromosomal inversion, heterochromatinization, and DNA methylation may be the underlying mechanisms of recombination suppression^51^. Large-scale heterochromatinization and loss-of-function regions are found in the Y chromosome of sorrel^52^. DNA methylation may defend against the insertion of DNA repeats derived from transposons and speed up heterochromatinization in specific regions of sex determination^53^. The methylation and heterochromatin levels of the male-specific DNA region of the Y chromosome are higher than those of the corresponding region of the X chromosome in *Papaya*^53^. The reduced recombination in sex chromosomes leads to the differentiation of their structure and function; male- or female-specific sequences accumulate in chromosomes, leading to a high degree of degeneration in sex chromosomes^51,54^. After a long period of evolutionary accumulation, autosomes may ultimately evolve into morphologically and functionally different sex chromosomes. In this study, we performed fine local mapping with SSR markers designed with sequences from the confined IESL of *S. triandra*, and severe recombination repression in the SDR was observed between the two sexes. Similar features are observed in *S. suchowensis*^22^, *S. viminalis*^23^ and *S. purpurea*^55^.

Dioecy has evolved hundreds of times from a hermaphroditic ancestor, and different genes may be involved in this process^56,57^, leading to great challenges in identifying the particular sex determination genes of different taxa of plants. The identification of sex-determining genes is of great significance to reveal the mechanism underlying sex determination in flowering plants. Therefore, genes involved in floral development located in the SDR are good putative candidates. Akagi et al.^58^ found that the *SyGI* gene, located in the Y-specific region, was involved in carpel development. In *Diospyros lotus* (XY system), the only identified sex-determining gene, *OGI*, which is located in a male-specific region, encodes 21-bp small RNAs targeting the autosomal gene *MeGI*, which acts as a maleness suppressor^59^.

In this study, a single SDR was physically located within a physical interval of 6.5 Mb in chromosome XV in *S. triandra*, which shows clear female heterogamety. This observation is consistent with findings in other willow species^22,23^. However, the estimated size of the SDR should not be considered definitive, due to the limited resolution of the genetic maps. In the confined IESL of *S. triandra*, six genes exhibit the molecular function of microtubule motor activity, which is involved in male reproductive development and function^60^. Similar gene groups are found in the *S. purpurea* SDR^55^. Another gene, *EVM0005130*, which contains a Myb-like DNA-binding domain, deserves special attention because it plays an important role in regulating anther and pollen development^61^. We also detected the interesting gene *EVM0045351*, containing a mitogen-activated protein kinase (MAP kinase) domain, which has been proposed to function as a regulator of both pollen development and germination^62^. Increasing numbers of studies have demonstrated that miRNAs play critical roles in regulating plant growth and stress responses as well as plant reproductive development. miRNA156 and 159, which are related to plant flower development, have been identified in the SDR.

In conclusion, the present study developed high-density linkage maps for *S. triandra*. The mapping of the sex locus revealed female heterogamety, indicating that sex in this willow species is determined through a ZW determination system. We confined the sex determination locus of *S. triandra* to a 6.5 Mb genomic region that harbors 249 genes and 22 miRNAs. The region contains several promising sex determination candidates, which are worthy of special attention in future studies.

## Supplementary information

Table S1

Figure S1

Figure S2

Figure S3
