# Supplementary material for: Fine mapping of the sex locus in Salix triandra confirms a consistent sex determination mechanism in genus Salix
Source: Hortic Res. 2020 May 1;7:64. doi: 10.1038/s41438-020-0289-1 (PMC7193568; doi:10.1038/s41438-020-0289-1)
Supplement: Supplementary file 1 — Table S1 [file 41438_2020_289_MOESM1_ESM.docx]

Table S1. The mapped ratio of RAD sequencing reads for each sample.

| Sample ID | Mapped Ratio（%） | Sample ID | Mapped Ratio（%） |
| --- | --- | --- | --- |
| 1 | 81.27 | 79 | 83.2 |
| 2 | 81.49 | 80 | 82.07 |
| 3 | 84.65 | 81 | 81.99 |
| 4 | 81.21 | 82 | 72.81 |
| 5 | 81.8 | 83 | 83.83 |
| 6 | 82.5 | 84 | 77.37 |
| 7 | 82.85 | 85 | 72.67 |
| 8 | 85.22 | 87 | 76.6 |
| 9 | 83.35 | 88 | 81.05 |
| 10 | 80.97 | 89 | 80.87 |
| 11 | 79.58 | 90 | 83.95 |
| 12 | 84.14 | 91 | 81.8 |
| 13 | 82.82 | 92 | 85.75 |
| 14 | 85.3 | 93 | 83.59 |
| 16 | 84.24 | 94 | 75.25 |
| 17 | 83.81 | 95 | 83.22 |
| 18 | 78.94 | 96 | 79.41 |
| 19 | 82.08 | 97 | 82.93 |
| 20 | 84.18 | 98 | 83.93 |
| 21 | 81.99 | 99 | 84.4 |
| 22 | 81.95 | 100 | 81.53 |
| 23 | 78.3 | 101 | 80.03 |
| 24 | 80.44 | 102 | 84.47 |
| 25 | 78.02 | 103 | 85.43 |
| 26 | 81.17 | 104 | 80.22 |
| 27 | 81.16 | 105 | 83.69 |
| 28 | 76.75 | 106 | 80.77 |
| 29 | 83.26 | 107 | 83.6 |
| 30 | 82.31 | 108 | 83.41 |
| 31 | 84.65 | 109 | 80.54 |
| 32 | 67.6 | 110 | 73.4 |
| 33 | 83.55 | 111 | 82.56 |
| 34 | 77.16 | 112 | 84.04 |
| 35 | 82.51 | 113 | 83.7 |
| 36 | 81.89 | 114 | 81.88 |
| 37 | 80.79 | 115 | 46.89 |
| 38 | 55.8 | 116 | 74.35 |
| 39 | 77.12 | 117 | 73.83 |
| 40 | 72.4 | 118 | 66.05 |
| 41 | 80.41 | 119 | 76.61 |
| 42 | 64.98 | 120 | 77.2 |
| 43 | 82.5 | 121 | 79.09 |
| 44 | 79.16 | 122 | 78.93 |
| 45 | 80.19 | 123 | 75.9 |
| 46 | 74.92 | 124 | 79.93 |
| 47 | 81.52 | 125 | 82.36 |
| 48 | 80.51 | 126 | 82.31 |
| 49 | 75.63 | 127 | 82.87 |
| 50 | 73.25 | 129 | 80.18 |
| 51 | 79.91 | 130 | 82.63 |
| 52 | 81.77 | 131 | 81.8 |
| 53 | 82.88 | 132 | 83.51 |
| 54 | 80.04 | 133 | 83.97 |
| 55 | 78.63 | 134 | 82.98 |
| 56 | 80.98 | 135 | 82.13 |
| 57 | 73.1 | 136 | 83.75 |
| 58 | 74.68 | 137 | 78.29 |
| 59 | 83.14 | 138 | 82.37 |
| 60 | 81.61 | 139 | 79 |
| 61 | 83.17 | 140 | 69.94 |
| 62 | 81.89 | 141 | 81.52 |
| 63 | 84.43 | 142 | 84.25 |
| 64 | 81.34 | 143 | 80.67 |
| 65 | 85.19 | 144 | 84 |
| 66 | 82.69 | 145 | 80.4 |
| 67 | 82.46 | 146 | 74.87 |
| 68 | 83.4 | 147 | 82.21 |
| 69 | 83.34 | 148 | 80.41 |
| 70 | 85.29 | 149 | 83.94 |
| 71 | 83.47 | 150 | 80.36 |
| 72 | 83.03 | 151 | 77.13 |
| 73 | 74.6 | 152 | 78.96 |
| 74 | 82.33 | 153 | 78.66 |
| 75 | 82.52 | 154 | 84.14 |
| 76 | 77.91 | 155 | 81.75 |
| 77 | 83.35 | DB134 | 88.04 |
| 78 | 83.15 | DB447 | 86.28 |
