# Supplementary figures and images for: Fine mapping of the sex locus in Salix triandra confirms a consistent sex determination mechanism in genus Salix
Source: Hortic Res. 2020 May 1;7:64. doi: 10.1038/s41438-020-0289-1 (PMC7193568; doi:10.1038/s41438-020-0289-1)

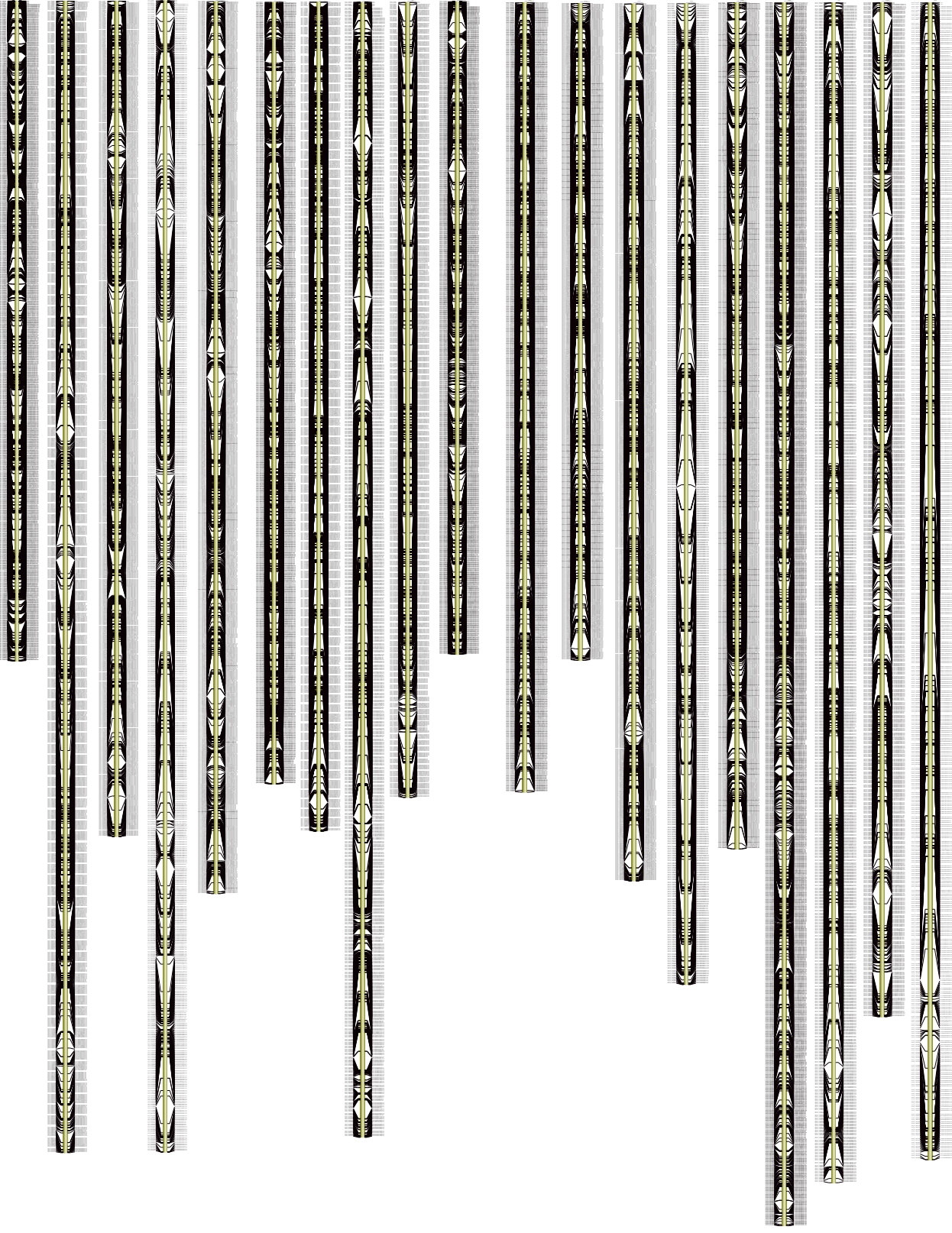

Supplement: Supplementary file 2 — Figure S1 [file 41438_2020_289_MOESM2_ESM.pdf]

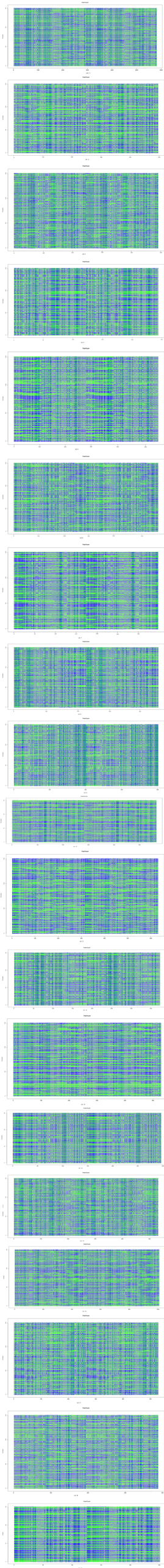

Supplement: Supplementary file 3 — Figure S2 [file 41438_2020_289_MOESM3_ESM.pdf]

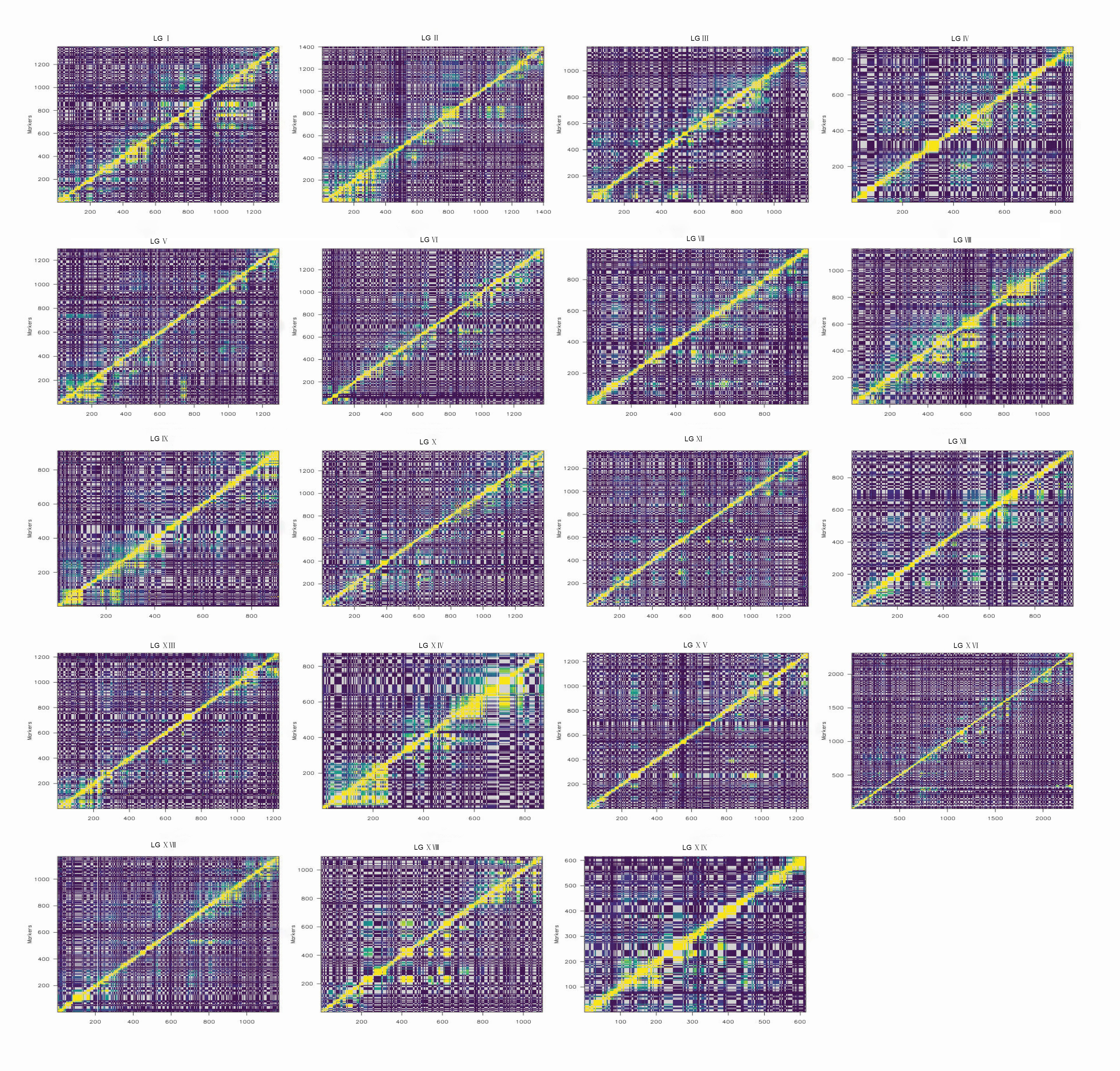

Supplement: Supplementary file 4 — Figure S3 [file 41438_2020_289_MOESM4_ESM.jpg]
